# Effects of low temperatures on quiescence in *Trichogramma evanescens* Westwood and *T. chilonis* Ishii reared on *Plodia interpunctella* (Hübner): implications for mass rearing

**DOI:** 10.1038/s41598-024-53702-z

**Published:** 2024-02-08

**Authors:** Md. Mahbub Hasan, M. Nishat Parvin, Christos G. Athanassiou

**Affiliations:** 1 Department of Zoology, Rajshahi University, Rajshahi, 6205 Bangladesh; 2 Laboratory of Entomology and Agricultural Zoology, Department of Agriculture, Crop Production and Rural Environment, University of Thessaly, Phytokou str., 38446 N. Ionia, Magnesia Greece

**Keywords:** Zoology, Entomology, Climate-change ecology

## Abstract

The egg parasitoids of the genus *Trichogramma* are important potential biological control agents for a wide range of lepidopteran pests. Cold storage of host eggs has been proposed as a valuable technique for ensuring the release of sufficient parasitoid numbers whenever it is needed. In this context, the impact of low temperatures to induce quiescence in *T. evanescens* Westwood and *T. chilonis* Ishii was studied using eggs of Indian meal moth *Plodia interpunctella* (Hübner). Prepupae of the parasitoids were stored for 15, 30, 45, 60 and 75 d at 4 °C, following a 7 d period of acclimation at 10 °C. Both parasitoid species seem to survive unfavorable temperature conditions by entering a state of quiescence. Parasitism, adult emergence, sex ratio and progeny quality were not affected by cold storage in either parasitoid species for up to 30 d of storage. Parasitized host eggs of *P. interpunctella* can be stored for up to 60 d at 4 °C for both parasitoids, but there was no emergence at 75 d. General productivity values gradually decreased as the duration of storage lengthened for both species. Our results clearly reveal that the eggs parasitized by these species can be stored for up to 30 d at 4 °C in a state of quiescence without much loss of their performance compared to the control eggs.

## Introduction

The egg parasitoid, *Trichogramma* spp. (Hymenoptera: Trichogrammatidae) are among the most exploited groups of parasitoids for biological control and integrated pest management^1^. To date, species of this genus have been considered in management programs for various insect pests on 18 crops and can either be explored as alternatives to chemical pesticides or as important components in integrated pest management (IPM) programs^1^. The most common species of this genus are *Trichogramma minutum* Riley, *T. evanescens* Westwood, *T. chilonis* Ishii, *T. pretiosum* Riley, *T. deion* Pinto and Oatman and *T. dendrolimi* Matsumura. These species have been found in an extremely large variety of tritrophic systems, and on different hosts^2^. The success of biological control programs is often related to problems and cost of rearing beneficial insects due to their relatively short shelf-life and difficulties in synchronizing parasitoid and host life cycles^1^. Dormancy management could offer opportunities to carefully time the supply of mass-reared insects upon demand and to enhance product quality^3^. Specifically, dormancy management could enable effective mass rearing of insects that require dormancy, the ability to stockpile and mobilize them upon demand, product shipment, and release. However, there is a clear gap in knowledge of the roles and mechanisms of dormancy on life cycle synchronization in natural enemies^3^. A period of dormancy interrupts the life cycle of many insect species, serving to protect them from harsh environmental conditions. Dormancy could occur in most of the parasitoids either by quiescence or diapause, conditions that can be further implemented to facilitate mass rearing protocols for biological control^3–6^. In quiescence, insect development is halted or slowed indirectly in response to unsuitable environmental conditions, while development resumes under favorable conditions, although this phenomenon varies among species^7–9^. The implementation of most suitable “parasitoid storage” methods is considered as a valuable tool to maintain high quality mass production and to allow synchronization of field releases during pest outbreaks^10,11^. In the recent years, cold storage of host eggs received more attention because of its significant role in biological control programs^12^. Moreover, it provides the availability in maximum numbers at the time of release, along with flexibility and quality assurance in mass production^13^. In general, storage temperatures ranging from 0 to 15 °C are widely used for parasitoids^10^; however, the optimal temperature depends upon the relative balance between the reduction of metabolic rates and the risk of accumulating chilling-related injury^11^. A gradual or rapid acclimation is usually desirable for pre-exposure to sublethal low temperatures^14–16^. Moreover, it has been reported that there is a positive impact on cold storage tolerance for acclimated parasitoids^17,18^ despite the fact that there might be some effects in decreasing tolerance as well^19^.

Cold storage of parasitoids is crucial for mass production, as the major outcome of this procedure is quality control of the exposed individuals^20–23^. In the case of species of the genus *Trichogramma*, storage can be achieved with or without previous diapause induction^24^. Smith^25^ reported that immatures of several *Trichogramma* species can enter diapause or quiescence within host eggs and thereby tolerate long periods of subfreezing temperatures. This approach could be important in establishing mass rearing of *Trichogramma* species and relative parasitoid applications.

The Indian meal moth, *Plodia interpunctella* (Hübner) (Lepidoptera: Pyralidae) is probably the most important stored product moth species globally, that is extremely destructive in a wide range of commodities, ranging from amylaceous products to dried fruits and nuts, causing serious losses^26,27^. Apart from its significance as a pest, *P. interpunctella* has been thoroughly utilized as a host for the rearing of several parasitoid species, that can be further used for biological control of several moths of economic importance^3,28,29^. Moreover, the diapause of this species, as well as its response to low temperatures, have been investigated in detail, often in terms of the synchronization of these parameters with parasitoid development and progeny production, towards the use of these factors in mass rearing protocols of certain parasitoid species^3,30^.

**Figure 1:**
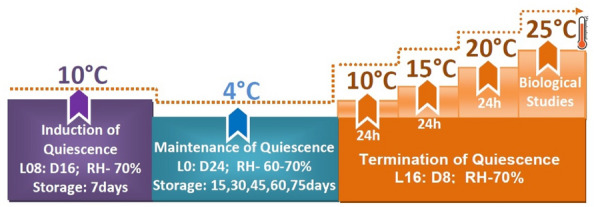
Schematic diagram of the experimental protocol.

In this context, the objective of the present study was to determine the effect of storage of parasitized eggs at low temperature for varying periods on key biological traits that are directly related to parasitism longevity in *T. evanescens* and *T. chilonis*, two egg parasitoids that have been thoroughly tested for the control of stored product species^31^. In this effort, we have utilized *P. interpunctella* as the host species (Fig. 1).

## Results

**Figure 2:**
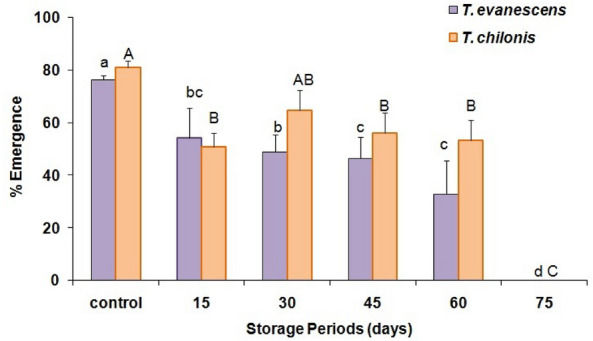
Effect of cold storage period on the percentage (mean ± SE) of adult emergence of *T. evanescens* and *T. chilonis* reared on *P. interpunctella* (Bars followed by the same letters are not significantly different; HSD test at 0.05; lowercase letters for *T. evanescens*, uppercase letters for *T. chilonis*).

**Figure 3:**
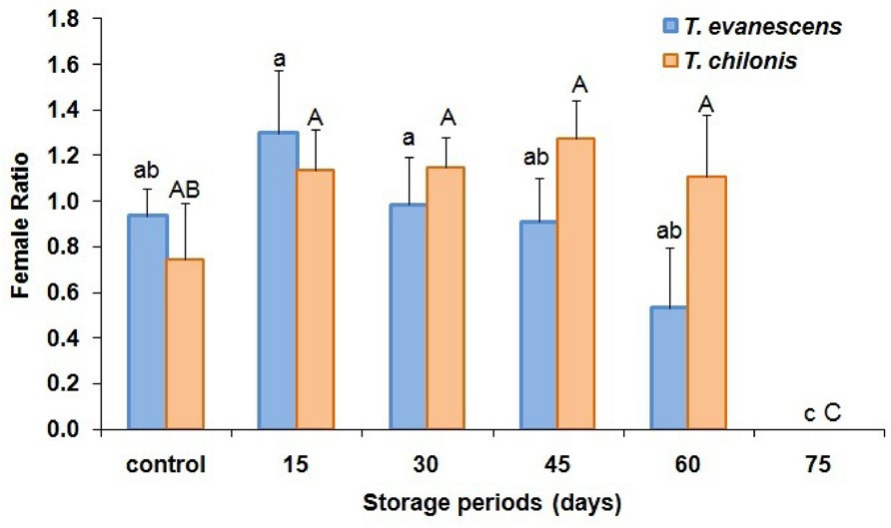
Effect of storage periods on the female ratio (males: females ± SE) in *T. evanescens* and *T. chilonis* reared on *P. interpunctella* (Bars followed by the same letters are not significantly different; HSD test at 0.05; lowercase letters for *T. evanescens*, uppercase letters for *T. chilonis*).

**Figure 4:**
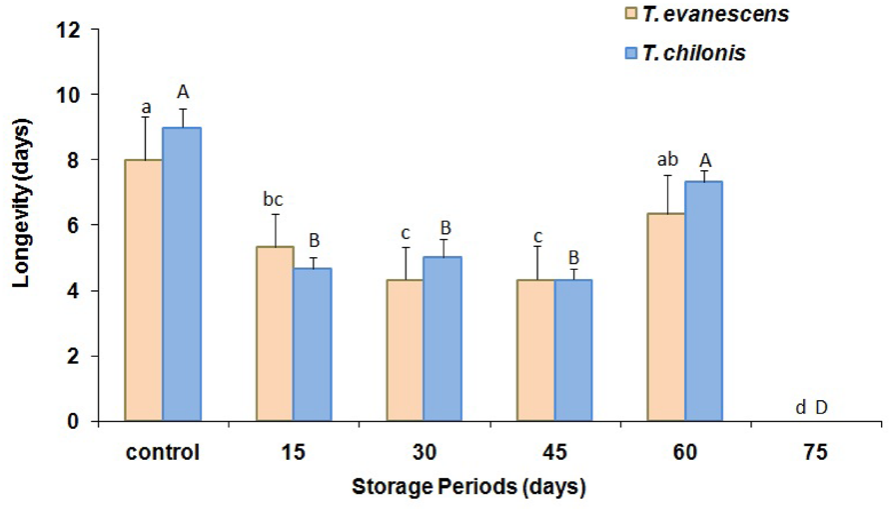
Effect of cold storage period on the adult longevity (mean ± SE) of *T. evanescens* and *T. chilonis* reared on *P. interpunctella* (Bars followed by the same letters are not significantly different; HSD test at 0.05; lowercase letters for *T. evanescens*, uppercase letters for *T. chilonis*).

The durations of cold stored parasitized eggs of *P. interpunctella* significantly affected the percent adult emergence in both *T. evanescens* (F = 45.3; df = 5.12; *P* < 0.01) and *T. chilonis* (F = 44.2; df = 5.12; *P* < 0.01). Moreover, there was also a significant difference between species in adult emergence (F = 20.5; df = 1.34; *P* < 0.001). In the control, the maximum percent of parasitoid emergence reached 76 and 81% for *T. evanescens* and *T. chilonis*, respectively, while the minimum emergence was 33% for the 60 d storage at 4 °C for *T. evanescens* (Fig. 2). However, no adult emergence was recorded from the 75 d cold storage periods for either species. Female sex-ratio resulting from the cold-stored host eggs varied significantly in *T. evanescens* (F = 5.1; df = 5.12; *P* = 0.01) and *T. chilonis* (F = 7.5; df = 5.12; *P* < 0.01) (Fig. 3). However, these ratios did not differ significantly between the two species (F = 1.2; df = 1.34; *P* = 0.28). Longevity of females significantly declined with increase of exposure of cold storage in *T. evanescens* (F = 56.0; df = 5.12; *P* < 0.01) and *T. chilonis* (F = 56.4; df = 5.12; *P* < 0.01), with the exception of 60 d (Fig. 4). The adults resulting from the cold-stored host eggs showed a shorter life span compared to control adults. Also, longevity did not differ significantly between the species produced from the cold stored host eggs (F = 2.1; df = 1.34; *P* = 0.16).

**Figure 5:**
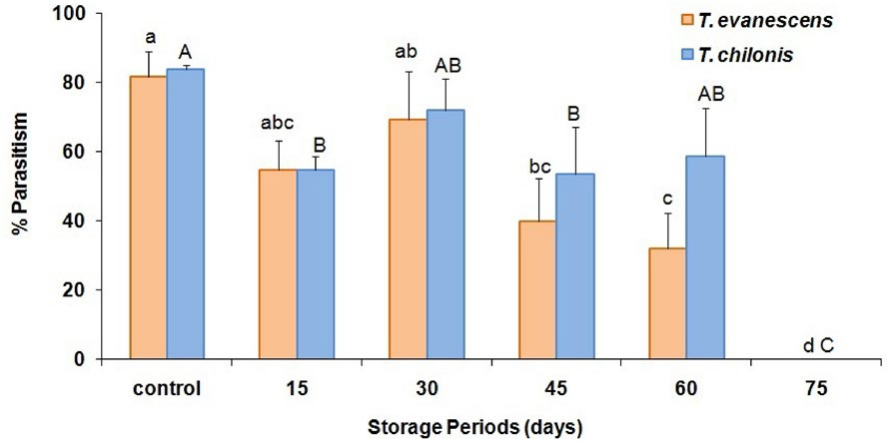
Effect of cold storage period on the percentage of parasitism (mean ± SE) of *T. evanescens* and *T. chilonis* reared on *P. interpuncella* (Bars followed by the same letters are not significantly different; HSD test at 0.05; lowercase letters for *T. evanescens*, uppercase letters for *T. chilonis*).

Parasitism percent of adult females in F_1_ progeny were higher in non-cold stored host eggs (control) for *T. evanescens* (81%) and *T. chilonis* (84%), as compared with the cold stored ones (Fig. 5). Results indicated that cold storage of host eggs had a significant effect on the F_1_ parasitism of *T. evanescens* (F = 21.2; df = 5.12; *P* < 0.01) and *T. chilonis* (F = 22.5; df = 5.12; *P* < 0.01) females in the F_1_ generation. We also observed that *T. chilonis* females resulting from all cold-stored eggs had a considerably higher percentage of parasitism except 15 d, as compared with *T. evanescens*, although, there were no significant differences (F = 4.0; df = 1.34; *P* = 0.06) between species in F_1_ parasitism. The percentage of parasitism decreased in adult females resulting from cold stored host eggs compared to non-cold stored ones (Fig. 5).

In general, key biological traits, such as fecundity (blackened eggs), adult emergence and ratio of females, of the parasitoid adults that emerged from host eggs that had been in cold storage were reduced as compared to controls (Table 1). The values of general productivity (GP) gradually decreased as duration of storage lengthened for both species (Table 1). However, parasitism efficacy (PE) was reduced up to 29.4 and 64.0 for *T. evanescens* and *T. chilonis*, respectively, at the 60d cold storage interval. Accordingly, the highest reduction in parasitism efficiency (RPE) reached up to 70.61and 36.03%, at 60 d cold storage groups for *T. evanescens* and *T. chilonis*, respectively (Table 1).

**Table 1:** General productivity (GP), parasitism efficiency (PE), and reduction in parasitism efficiency (RPE) of *T. evanescens* and *T. chilonis* reared on *P. interpunctella* eggs that had been stored at 4 °C for different periods.

Species	Cold storage periods (days)	GP	PE	RPE
*T. evanescens*	Control	8.47	–	–
	15	7.36	86.92	13.08
	30	6.84	80.79	19.21
	45	3.91	46.13	53.87
	60	2.49	29.39	70.61
	75*	–	–	–
*T. chilonis*	Control	7.82	–	–
	15	7.73	91.30	8.70
	30	7.52	88.81	11.19
	45	5.74	67.74	32.26
	60	5.42	63.97	36.03
	75*	–	–	–

*There was no adult emergence.

## Discussion

The results of the current study clearly suggest that cold storage of *P. interpunctella* eggs for 30 d is viable storage duration for production of increased quantities of *Trichogramma* parasitoids. At the same time, this storage also maintains the “quality” of the parasitoids, i.e. their ability to provide satisfactory progeny production and the concomitant increased parasitism rates. Our study shows that storing *P. interpunctella* eggs at 4 °C for more than 60d, negatively affects the overall survival, and should be avoided.

It is not clear if the exposed individuals here entered diapauses or the experimental protocol followed here caused quiescence, given that quantitative data were not collected towards this direction. This direct response to non-suitable environmental conditions, and the subsequent response with the return of the favorable conditions, i.e. increase of temperature and host availability, may mostly suggest quiescence rather than diapause^3,6^. Andreadis et al.^32^ found that the parasitoid was generally more susceptible than the host while following a similar protocol with low temperature or the exposure of the endoparasitoid *Venturia canescens* (Gravenhorst) (Hymenoptera: Ichneumonidae) on larvae of the Mediterranean flour moth, *Ephestia kuehniella* Zeller (Lepipdoptera: Pyralidae). However, in that study, the age of the parasitoid played a critical role in its survival after exposure to low temperature, which can be taken into account to reduce mortality in cases of low temperature parasitoid preservation^32^ In present study, we did not measure survival of the host, in terms of *P. interpunctella* egg hatch, but our data show that both *T. evanescens* and *T. chilonis* were able to survive long periods at low temperature. The percentages of emergence that were generally lower than those of individuals that were not exposed to cold, suggesting that even an exposure as short as 15 d can cause some parasitoid morality. It is generally considered that exposure, and thus preservation from a practical point of view, of parasitoids at low temperature may be subjected to considerable fitness costs, including increased mortality^11,33^, which stands in accordance with the present findings. However, Cagnotti et al.^34^ reported that *Trichogramma nerudai* Pintureau prepupae, that had been stored at suboptimal temperature (12 ± 0.5 °C) were not affected when the storage was as short as 10 or 20d. In this context, preconditioning may play a positive role in the duration of the parasitoid preservation^3,34^.

Despite the fact that there might be some mortality after exposure to cold, we found that these losses are partially compensated by specific adaptations that were not expected, such as changes to male: female ratios, for both species, and progeny production attributes that are similar to those of the non-acclimated individuals. This is expressed more vigorously in the case of *T. chilonis*, which generally performed better than *T. evanescens*. Interestingly, despite the fact that exposure to cold reduced longevity of both species for exposure periods of 15, 30 and 45 d, at 60 d longevity was increased to levels that were comparable to controls. This may be attributed to the initiation of a cold-hardening procedure that might have been triggered at these conditions, switching eventually the adaptation of the parasitoids. The increased adaptation, through improved cold-hardening that is exposed more vigorously in long exposures, has been also identified in other parasitoid species^30,32^.

For the endoparasitoid *Microplitispro deniae* Rao and Chandry (Hymenoptera: Braconidae), Yan et al.^35^ found that there were no subsequent effects of exposure to cold of the parental individuals during the next two generations. Similar results have been also reported by Silva et al. ^36^ for the aphid parasitoid *Diaeretiella rapae* (McIntosh) (Hymenoptera: Braconidae). It is well established that exposure to cold is likely to cause some major, and in most cases irreversible, negative changes in fecundity of the exposed parasitoids, which, in turn, may seriously affect their performance after the termination of the exposure^37,38^. For instance, Colinet and Hance^39^ found that exposure to suboptimal temperatures seriously affected the reproductive system of the aphid parasitoid *Aphidius colemani* (Viereck) (Hymenoptera: Braconidae). In accordance with what has been reported by Yan et al.^33^ for *M. prodeniae*, parasitism rates of the offspring that were produced by the exposed parental adults were not seriously affected, suggesting that any effect is physiologically-related and not transferable to the next generation. This is particularly important in developing strategies for cold storage of parasitoids, and most studies suggest that there is no carryover to the subsequent generations that will be produced by the exposed parental individuals. Still, our work is in accordance with previous reports considering the negative effect of exposure to low temperature to progeny production capacity, demonstrated in both *T. evanescens* and *T. chilonis*. Paradoxically, we found that the relationship between parasitism rates and duration of exposure to cold was not linear, and, to some extent, gave dissimilar results. While for *T. evanescens* exposure for 15 and 30 d gave similar parasitism results with the controls, longer exposures was subsequently reduced to levels that were not comparable to those of the controls. On the other hand, for *T. chilonis*, parasitism caused by parasitoid adults that had been exposed for 15 d was lower than that in the controls, but at longer exposures did not differ significantly from the controls. All the above suggest the potential occurrence of specific physiological responses that are exhibited only at specific temperature-exposure combinations, and merit additional investigation. However, our results can be also related to the effect of low temperature on *P. Interpunctella* eggs, a feature that might have indirectly affected survival and performance of the parasitoids. In this context, Athanassiou et al.^30^ found that eggs of *P. interpuntella* were particularly susceptible to cold, and were much more susceptible than larvae, in contrast with most stored product insects where the egg is by far the most cold-tolerant life stage^31,40–42^. The exposure of eggs to cold may cause temporal physical damage to host eggs, that subsequently influences the overall parasitoid performance.

The results of our work clearly show that there are specific intervals of cold storage during which the parasitoids can remain unaffected for relatively long periods of time, which here can reach 60 d, if the storage temperature is + 4 °C. Although we observed some adverse effect in longevity and parasitism rates, the technique described here can be further utilized in mass rearing strategies of egg parasitoids for relatively long periods that will allow shipment and application in biological control programs. The cost of cold storage of parasitoids should be further analyzed in comparison with the cost of other storage methods that are being usually practiced.

## Methods

### Parasitoid rearing

The parasitoids *T. evanescens* and *T. chilonis* were originally received from the Bangladesh Agricultural Research Institute (BARI), Gazipur and Sugarcane Research Institute, Ishwardi, Bangladesh. These species were cultured and mass-reared using eggs (< 24 h old) *P. interpunctella*, at the Post-Harvest Entomology Laboratory, Department of Zoology, Rajshahi University, as suggested by Hegazi et al.^43^*.* The host eggs were pasted on paper strips (2 × 10 cm) with gum arabic glue (Daler Rowney Co., Cardiff, UK) which helps to easy handling as well as counting and then exposed to the parasitoids in glass jars (2L) supplied with cotton swabs soaked in honey solution (10% v/v) for adult diet^44^. The jars were covered by cloth-wrapped cotton fixing with rubber bands. The egg strips were changed daily to minimize super-parasitism.

### Host rearing

The culture of *P. interpunctella* was maintained at the Post-Harvest Entomology Laboratory, Department of Zoology, Rajshahi University, Bangladesh. They were reared on a standardized diet as suggested by Phillips and Strand^45^. The parasitoids and host cultures were maintained in an incubator set at 27 ± 0.5 °C, 70% relative humidity (RH), and a photoperiod of 16:8 (L: D) h^29^.

### Experimental setup for cold storage

Mixed aged (1–2 d old) *T.evanescens* and *T. chilonis* adults were kept separately in glass tubes (84 × 2.5 cm) containing host egg paper strips (2.5 × 10 cm) for 24 h at 25 °C, 16:8 (L:D) h and 60 to 70% RH^46^. The host eggs were pasted on paper strips with gum arabic glue, and exposed to either of the species. The glass tubes were provided with cotton swabs soaked in 10% honey solution for adult diet. After exposure for 24 h, 50 paper strips containing parasitized eggs for each species were collected and transferred to acclimation conditions of 10 °C, 8:16 (L:D) and 70% RH, for 7 d. After that, the parasitized egg (blackened color) paper strips were transferred to an incubator set at 4 °C, 24 h dark and 60–70% RH for 15, 30, 45, 60, and 75 d^23^. Five replicates, having fifty parasitized eggs per strip for each parasitoid were carried out per treatment. After completing the desired storage periods, the egg strips were removed from each treatment and kept at 10, 15 and 20 °C for 24 h at each temperature. Finally, the egg strips were shifted to an incubator set at 25 °C, 16:8 (L:D) and 70% RH until adult emergence. The experimental setup for cold storage is summarized in the schematic diagram of Fig. 1. After that, the percent emergence for each parasitoid was recorded. The sex-ratio (female ratio) and longevity of adults were also recorded, along with the efficiency of parasitism of cold stored adult parasitoids on fresh eggs.

The number of parasitized eggs (blackened host eggs) was considered as the fecundity of the females in each cold storage exposure. The adult emergence percentage was calculated as (number of emerged adults divided by the number of parasitized eggs) × 100. The general productivity (GP) was estimated as GP = rate of emergence × rate of produced females in progeny × fecundity^47^, and reduction in parasitism efficiency (RPE) was determined relative to control as RPE = [(GP of control batch − GP of individual storage exposure)/GP of control batch] × 100 while parasitism efficiency (PE) was calculated as PE = (GP of individual storage exposure/GP of control batch) × 100.

### Statistical analysis

The validation of the data distribution of each measured variables was carried out using Shapiro–Wilk’s test. The data were arcsine transformed before the analysis (untransformed data are presented in the Figs. and Tables). Then, all data were analyzed through ANOVA using the SAS software (PROC.GLMMIX)^48^, separately, for each of the trait including parasitism rates, percent emergence, sex-ratios and adult longevity. Means were compared by Tukey–Kramer (HSD) test at 5%.

### Ethical approval

In the frame of this study, no experiments have been conducted on animals or humans.

## Data Availability

The datasets analyzed during the current study available from the corresponding author on reasonable request.
